# Micro-flow synthesis and structural analysis of sterically crowded diimine ligands with five aryl rings

**DOI:** 10.3762/bjoc.9.268

**Published:** 2013-11-01

**Authors:** Shinichiro Fuse, Nobutake Tanabe, Akio Tannna, Yohei Konishi, Takashi Takahashi

**Affiliations:** 1 Department of Applied Chemistry, Tokyo Institute of Technology, 2-12-1, Ookayama, Meguro-ku, Tokyo, 152-8552, Japan; 2 Mitsubishi Chemical Croup, Science and Technology Research Center, Inc., 1000 Kamoshida-cho, Aoba-ku, Yokohama 227-8502, Japan

**Keywords:** amidine formation, diimine, flow chemistry, polymerization

## Abstract

Sterically crowded diimine ligands with five aryl rings were prepared in one step in good yields using a micro-flow technique. X-ray crystallographic analysis revealed the detailed structure of the bulky ligands. The nickel complexes prepared from the ligands exerted high polymerization activity in the ethylene homopolymerization and copolymerization of ethylene with polar monomers.

## Introduction

**Figure 1:**
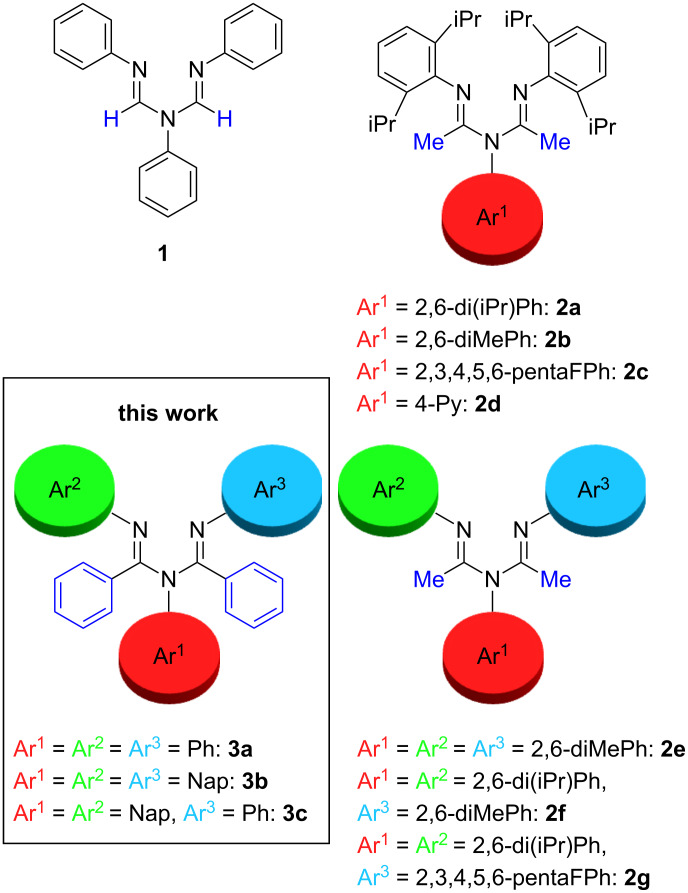
Sterically crowded, and neutral chelating diimine ligands.

The design of a ligand is a key step in the development of new catalysts because the ligand framework influences the reactivity of the metal center. That is why sterically crowded and neutral-chelating diimine ligands have garnered a great deal of attention [1–17]. In recent years, *N*-aryl 1,3,5-triazapenta-1,4-dienes **1** and **2** have been reported, and they are useful with late transition metal olefin-polymerization catalysts [18–19], and for the stabilization and isolation of reactive metal species [20–21]. In 1997, Murillo and coworkers reported the synthesis of a neutral, and bulky chelating ligand **1**, and its use in the formation of a Co complex (Figure 1) [22]. Stephan and coworkers reported the synthesis of a bulkier chelating ligand **2a**, and its use in the formation of various metal complexes [20–21]. Rojas and coworkers reported the synthesis of a series of bulky chelating ligands **2b–g**, and detailed their use in the preparation of ethylene polymerization catalysts [18–19]. We became curious about the structure and function of 1,2,3,4,5-pentaaryl-1,3,5-triazapenta-1,4-diene ligand **3**, which is sterically more hindered, because there are as many as five aryl rings that can provide further opportunities to change and tune the steric and electrical environments of the ligands [23–24]. However, as far as we could ascertain, this has been reported only once [25]. In this pioneering work, 1,2,3,4,5-pentaphenyl-1,3,5-triazapenta-1,4-diene ligand **3a** was prepared, but the report included neither the complexation nor a detailed structural study.

Herein, we report an efficient micro-flow synthesis and structural analysis of sterically crowded 1,2,3,4,5-pentaaryl-1,3,5-triazapenta-1,4-diene ligands **3b** and **3c**, and their use in the copolymerization of ethylene and polar monomers. According to the previous report, 1,2,3,4,5-pentaphenyl-1,3,5-triazapenta-1,4-diene ligand **3a** was prepared by the reaction of *N,N′*-diphenyl benzamidine and *N*-phenylbenzimidochloride in benzene in 13 days [25]. In the present study, we intended to prepare these ligands from readily available materials in only one step [18,21].

## Results and Discussion

**Scheme 1:**
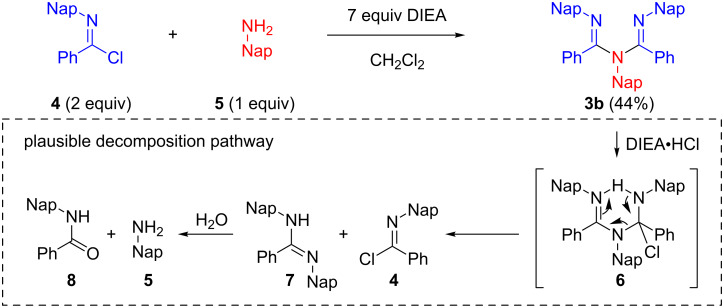
One-step synthesis of the ligand **3b**, and a plausible decomposition pathway for **3b** to naphthylamine (**5**) and amide **8**.

**Figure 2:**
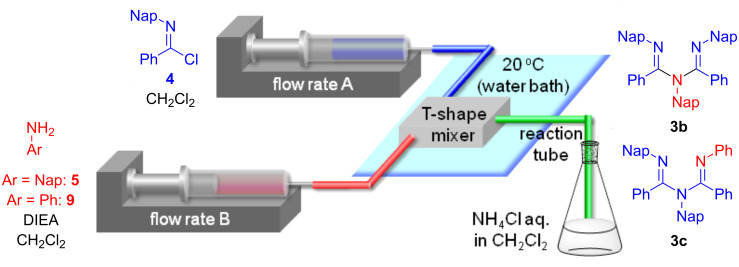
Micro-flow synthesis of the ligands **3b** and **3c**.

Two equivalents of *N*-naphthylbenzimidochloride (**4**) was reacted with one equivalent of naphthylamine (**5**) in CH_2_Cl_2_ in the presence of DIEA (*N,N*-diisopropylethylamine) at room temperature (Scheme 1). The reaction proceeded smoothly, and consumption of naphthylamine was confirmed by TLC analysis within 10 min. After an aqueous workup, the desired product **3b** was obtained in a moderate yield (44%) with the concomitant generation of naphthylamine (**5**) and amide **8**. Reportedly, **3a** can form an HCl adduct, and the adduct decomposes to the corresponding amidine and imidochloride [25]. It is conceivable that **3b** overreacted with DIEA·HCl to afford **6**, or **4** and **7** in the reaction mixture and that **5** and **8** were generated from the hydrolysis of these compounds under aqueous workup conditions. We decided to use a micro-flow reactor in order to suppress the overreaction [26–27] because the micro-flow technique [28–35] enables the precise control of reaction time and temperature. The micro-flow system was made from simple and inexpensive laboratory instruments (syringes, syringe pumps, water bath, T-shape mixer, standard tubing and fittings), as shown in Figure 2. The T-shape mixer was made of stainless steel and immersed in a water bath (20 °C). A solution of **4** (0.1 M) in CH_2_Cl_2_, a solution of aryl amine **5** or **9** (0.1 M), and DIEA (0.7 M) in CH_2_Cl_2_ were introduced using syringe pumps at the indicated flow rates. The reaction was quenched by pouring the mixture into a saturated aqueous solution of NH_4_Cl in CH_2_Cl_2_. After an aqueous workup, the products **3b** and **3c** were purified by silica gel chromatography. Reaction time was controlled by changing the flow rates.

As expected, the yield of **3b** was improved by reducing the reaction time (Table 1, entries 1–4). The highest yield was observed for a reaction time of 38 s (Table 1, entry 4). A further shortening of the reaction time resulted in a reduction in the yield because of substrate recovery (Table 1, entry 5).

**Table 1:** Micro-flow synthesis of ligands **3b** and **3c**.

entry	flow rate A [μL/min]	flow rate B [μL/min]	time [s]	Ar–NH_2_	yield^a^ [%]

1^b^	54	27	300	**5**	65
2^b^	106	53	150	**5**	72
3^b^	214	107	75	**5**	75
4^b^	426	213	38	**5**	84
5^b^	854	427	19	**5**	66
6^c^	214	107	50	**9**	55
7^c^	426	213	25	**9**	69
8^c^	854	427	13	**9**	66

^a^Isolated yield. ^b^Reaction tube volume is 400 μL. ^c^Reaction tube volume is 266 μL.

**Figure 3:**
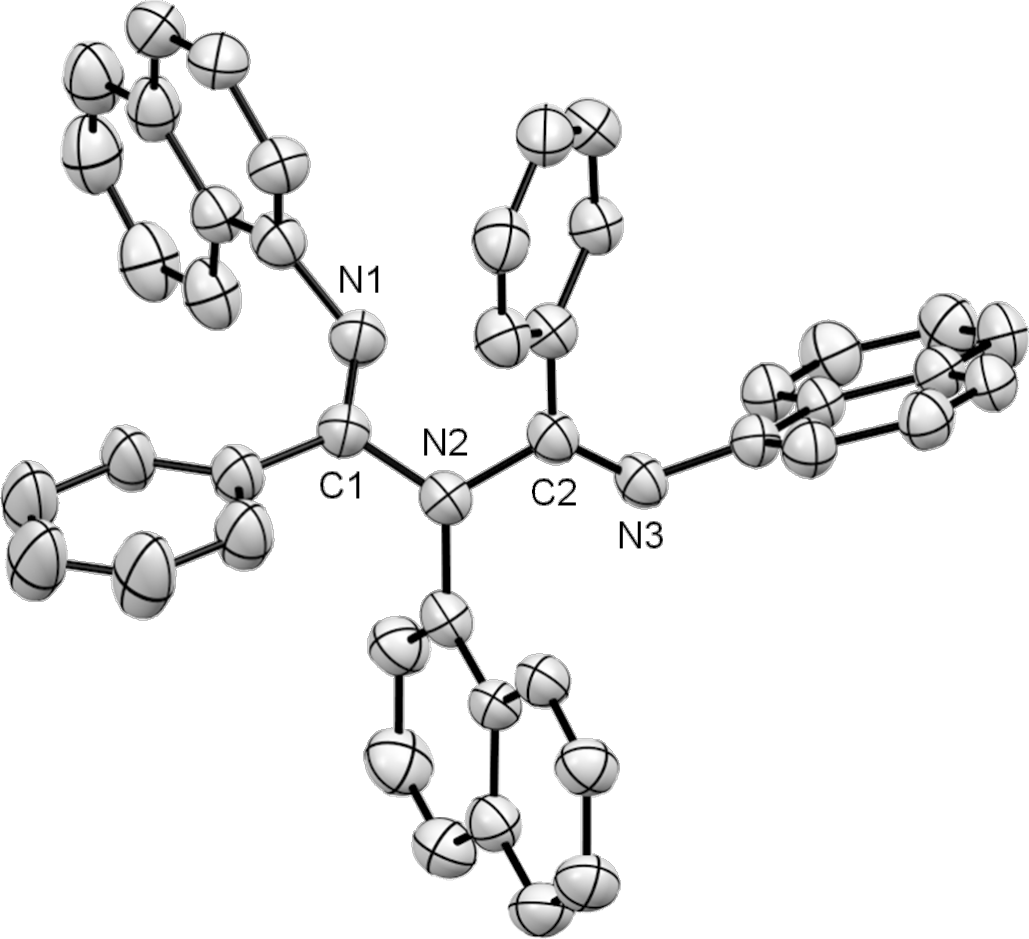
ORTEP drawing of **3b**. Thermal ellipsoids are set at 35% probability level. Hydrogen atoms are omitted for clarity. Selected bond lengths [Å] and angles [°] are as follows: C(1)–N(1) 1.273(4), C(1)–N(2) 1.420(4), C(2)–N(2) 1.421(4), C(2)–N(3) 1.278(4), N(1)–C(1)–N(2) 115.3(3), C(1)–N(2)–C(2) 117.0(3), N(2)–C(2)–N(3) 117.8(3).

The structure of the ligand **3b** was unambiguously determined by ^1^H NMR, ^13^C NMR, IR, HRMS and X-ray crystallographic analysis [36] (Figure 3). The ORTEP structure of **3b** showed that in the solid state the ligand adopts a non-planar arrangement similar to the previously reported ligands **2a–c**, and **2e**–**g** [18–19,21]. In ligand **3b**, N(1) and N(2) nearly occupied a common plane with C(1) and C(2), while N(3) was twisted out of this plane and was nearly perpendicular. The bond lengths for imines C(1)–N(1) and C(2)–N(3) were 1.273(4) and 1.278(4) Å, respectively, while the bond lengths for amines C(1)–N(2) and C(2)–N(2) were 1.420(4) and 1.421(4) Å, respectively. Reportedly, the two amine bond lengths were different in the case of ligands, **2a**, **2b**, **2e**, **2f**, and **2g** for which N=C–N–C=N was not in a common plane. On the other hand, the two amine bond lengths were nearly identical in the case of ligand **2c** where N=C–N–C=N was in a common plane. Interestingly, in the case of ligand **3b**, the bond lengths of the two amines, C(1)–N(2) and C(2)–N(2) were nearly identical, although N=C–N–C=N was not in a common plane.

**Scheme 2:**
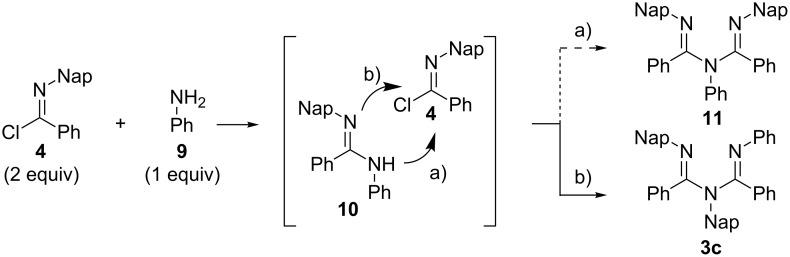
Synthesis of the ligand **3c** through the coupling of **4** and **9**.

The asymmetric ligand **3c** was obtained by the coupling of *N*-naphthylbenzimidochloride (**4**) with aniline (**9**). The product was obtained in a satisfactory yield (69%) under the conditions of entry 7 (25 s), as shown in Table 1. We speculated that the slightly lower yield of **3c** compared to **3b** came from the instability of **3c** during purification process. The compound **3c** was less stable than **3b**. Rojas et al. reported that the regioselectivity in the nucleophilic addition of an amidine to an imidochloride depends on the employed reaction conditions, – in particular, the order of addition and the base selection [37] – and the symmetric ligands were obtained through the coupling of *N*-2,6-di(iPr)-phenylbenzimidochloride with aryl amines in the presence of Et_3_N in toluene [18–19]. Interestingly, in our case, only the asymmetric ligand **3c** was obtained, although similar reaction conditions were employed (Scheme 2). The result showed that the nucleophilic addition of amidine **10** occurred from the sterically more hindered nitrogen atom (path b).

**Figure 4:**
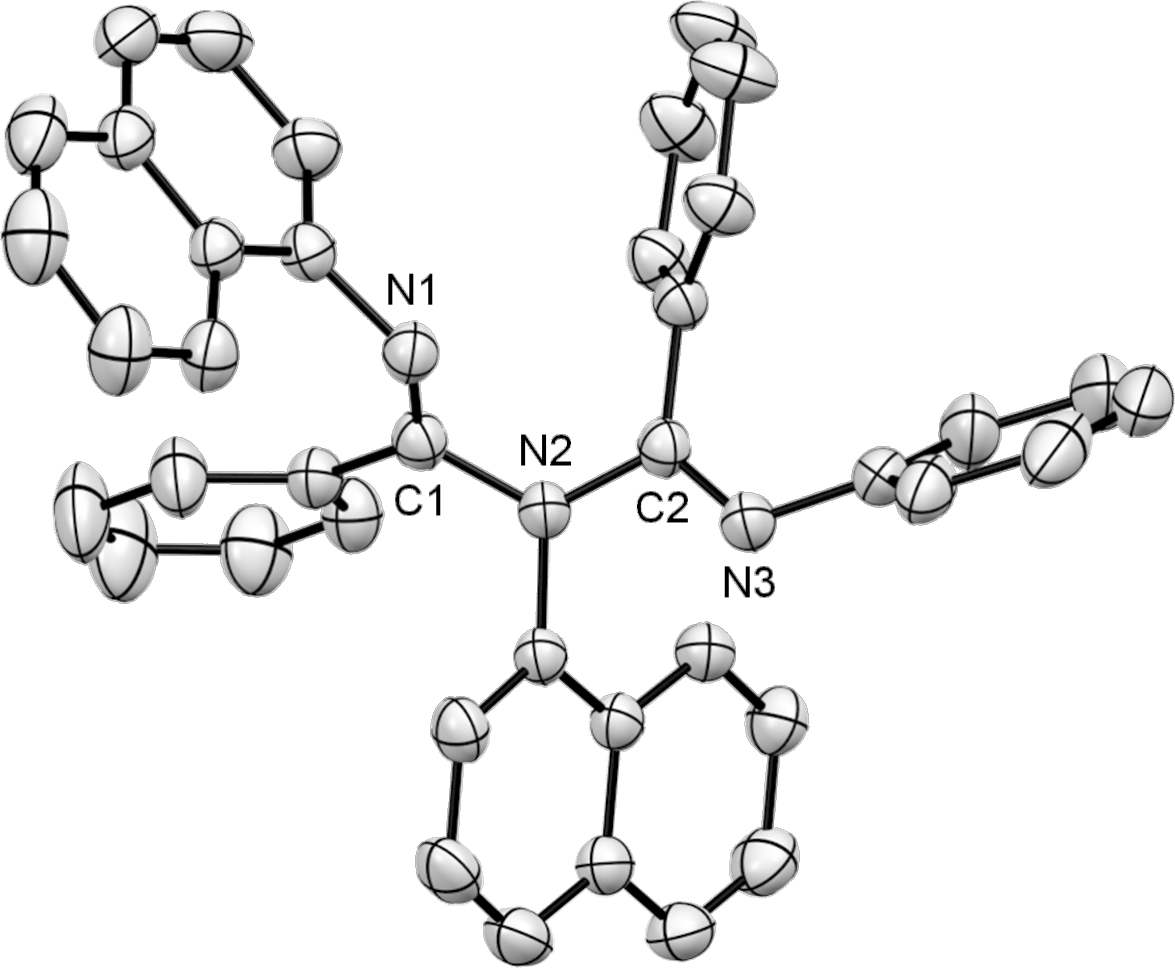
ORTEP drawing of **3c**. Thermal ellipsoids are set at 35% probability level. Hydrogen atoms are omitted for clarity. Selected bond lengths [Å] and angles [°] are as follows: C(1)–N(1) 1.269(3), C(1)–N(2) 1.413(3), C(2)–N(2) 1.411(3), C(2)–N(3) 1.274(3), N(1)–C(1)–N(2) 116.5(2), C(1)–N(2)–C(2) 119.1(2), N(2)–C(2)–N(3) 117.4(2).

The structure of the asymmetric ligand **3c** was unambiguously determined by ^1^H NMR, IR, HRMS and X-ray crystallographic analysis (Figure 4). The ORTEP structure of **3c** showed that in the solid state, the ligand adopted a non-planar arrangement similar to that of **3b**. In the ligand **3c**, N(1) and N(2) occupied a plane that was near that of C(1) and C(2), while N(3) was twisted out of this plane and was almost perpendicular. The bond lengths for imines C(1)–N(1) and C(2)–N(3) were 1.269(3) and 1.274(3) Å, respectively, while the bond lengths for amines C(1)–N(2) and C(2)–N(2) were 1.413(3) and 1.411(3) Å, respectively. The bond lengths for amines C(1)–N(2) and C(2)–N(2) were nearly identical, although N=C–N–C=N was not in a common plane. These features were similar to that of **3b**.

**Scheme 3:**
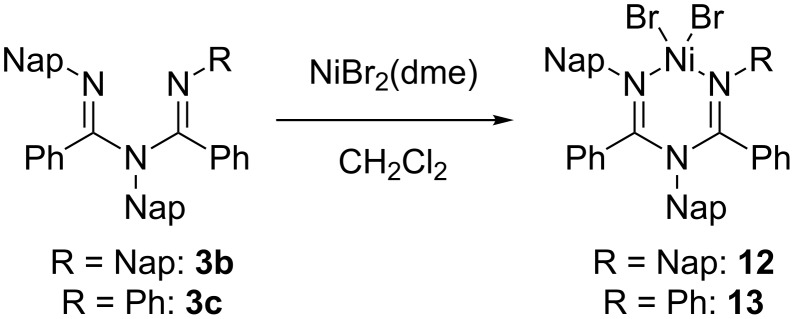
Complexation of the ligands **3b** and **3c** with NiBr_2_(dme).

**Figure 5:**
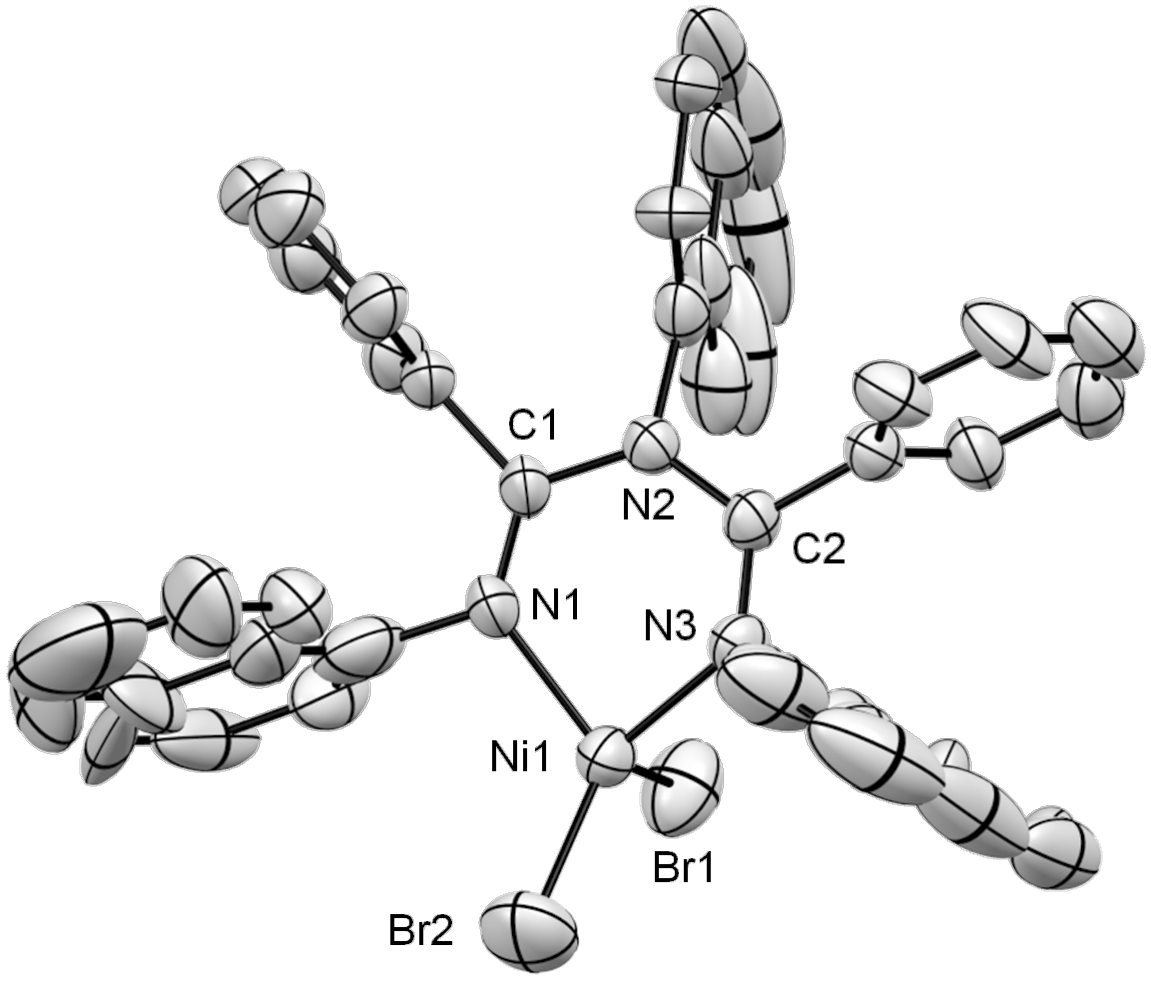
ORTEP drawing of **12**. Thermal ellipsoids are set at 35% probability level. Hydrogen atoms and cocrystallized CH_2_Cl_2_ are omitted for clarity. Selected bond lengths [Å] and angles [°] are as follows: C(1)–N(1) 1.277(11), C(1)–N(2) 1.393(11), C(2)–N(2) 1.396(11), C(2)–N(3) 1.305(11), Ni(1)–N(1) 1.947(8), Ni(1)–N(3) 1.943(8), N(1)–C(1)–N(2) 122.8(8), C(1)–N(2)–C(2) 126.2(7), N(2)–C(2)–N(3) 121.2(8), C(2)–N(3)–Ni(1) 127.5(6), N(1)–Ni(1)–N(3) 89.3(3), C(1)–N(1)–Ni(1), 127.7(6), N(1)–Ni(1)–Br(1) 102.2(3), N(1)–Ni(1)–Br(2) 120.9(3).

A complexation of the synthesized ligands **3b** and **3c** with nickel(II) was performed (Scheme 3) in accordance with a reported procedure [38]. An equimolar amount of NiBr_2_(dme) (dme = 1,2-dimethoxyethane) and the synthesized ligands were mixed in CH_2_Cl_2_ and stirred for 4 h at room temperature. Free ligands **3b** or **3c** were not observed by ^1^H NMR analysis of the obtained crude mixtures. NMR characterization of the complexes **12** and **13** was poor due to the paramagnetic nature of the pseudo-tetrahedral nickel centers. In the case of **12**, green needle-like crystals suitable for X-ray crystallographic analysis were obtained (Figure 5). The molecular structure confirmed the formation of a six-membered chelate ring by the ligand in a *N,N* binding mode to the nickel dibromide. All the aryl rings were almost perpendicular to the chelate plane probably due to the strong steric repulsions among the aryl rings. The tetrahedral geometry around nickel was distorted similar to the previously reported nickel complexes of **2b**, **2f** and **2g** [18–19]. For example, Br(1) was almost perpendicular to the plane of the metal-containing ring. This can be observed in the corresponding angle N(1)–Ni(1)–Br(1) 102.2(3)°, while the angle of N(1)–Ni(1)–Br(2) was 120.9(3)°. The C(1)–N(1), C(2)–N(3), C(1)–N(2) and C(2)–N(2) bond lengths were 1.277(11) Å, 1.305(11) Å, 1.393(11) Å and 1.396(11) Å respectively, indicating the double and single bond character around the imines and amine nitrogen, respectively.

A series of olefin polymerizations were briefly tested as shown in Table 2. Both catalysts **12** and **13** exerted high activity for the ethylene homopolymerization (Table 2, entries 1 and 2). As far as we could ascertain, neither propene homopolymerization nor copolymerization of ethylene with polar monomers by using the catalysts derived from *N*-aryl-1,3,5-triazapenta-1,4-dienes **1–3** have been reported [18–19]. Thus, we tested the polymerization activity of the catalyst **12** in the copolymerization of ethylene with 5-norbornen-2-ol (NBO) and ethyl acrylate (EtA) (Table 2, entries 3 and 4) and propene homopolymerization (Table 2, entry 5). Although the propene homopolymerization activity of catalyst **12** was moderate, catalyst **12** exerted a high activity in the copolymerization of ethylene with both polar monomers.

**Table 2:** Evaluation of polymerization activities of nickel complexes **12** and **13**.^a^

entry	catalyst^b^	monomer^c^	temperature, time [°C, h]	*V*_p_ [kg/mol·h]

1	**12**	ethylene	60, 0.5	140
2	**13**	ethylene	60, 0.5	110
3	**12**	ethylene/NBO	60, 1	25
4	**12**	ethylene/EtA	60, 1	35
5	**12**	propene	60, 1	5

^a^Entries 1–4 were carried out in a 2 L autoclave reactor in 1 L toluene in the presence of 500 equiv of modified methyl aluminoxane (MMAO)[39] as cocatalyst and 400 psig of ethylene. Entry 5 was carried out in a 2 L autoclave reactor in 750 mL propene in the presence of 500 equiv of MMAO as the cocatalyst. ^b^Entries 1, 4 and 5 were carried out using 0.11 mmol of the catalyst. Entries 2 and 3 were carried out using 0.10 and 0.09 mmol of the catalysts, respectively. ^c^Entries 3 and 4 were carried out using 500 equiv of NBO or EtA.

## Conclusion

In summary, sterically crowded diimine ligands **3b** and **3c** were prepared in one step in good yields using a micro-flow technique. One of the advantages of using microreactors is the ease of scale-up. It should be possible to scale-up our developed process by either continuous running or by increasing the number of the microreactors. X-ray crystallographic analysis revealed the detailed structure of ligands **3b** and **3c**. Interestingly, **3c** retained an asymmetric structure which ran contrary to a previous report. Unexpectedly, both bond lengths of the two amines C(1)–N(2) and C(2)–N(2) in both ligands were nearly identical, although N=C–N–C=N was not in a common plane. The complexation of **3b** and **3c** with nickel afforded **12** and **13**. X-ray crystallographic analysis of **12** revealed that all the aryl rings are nearly perpendicular to the chelate plane. The nickel complex **12** exerted a high polymerization activity in both ethylene homopolymerization and the copolymerization of ethylene with polar monomers. The synthesized ligands **3b** and **3c** retained as many as five aryl rings, which offered another opportunity to change the steric and electric environment. The developed process should be valuable for the preparation of various 1,2,3,4,5-pentaaryl-1,3,5-triazapenta-1,4-diene ligands and for the creation of novel and useful catalysts.

## Experimental

### General

NMR spectra were recorded on a JEOL Model ECP-400 (400 MHz for ^1^H, 100 MHz for ^13^C) instrument in the indicated solvent. Chemical shifts are reported in units of parts per million (ppm) relative to the signal (0.00 ppm) for internal tetramethylsilane for solutions in CDCl_3_ (7.26 ppm for ^1^H, 77.0 ppm for ^13^C). Multiplicities are reported by using the following abbreviations: s, singlet; d, doublet; t, triplet; q, quartet; m, multiplet; br, broad; and, *J*, coupling constants in Hertz. IR spectra were recorded on a Perkin-Elmer Spectrum One FTIR spectrometer. HRMS (ESI–TOF) was measured with a Waters LCT Premier^TM^ XE. All reactions were monitored by thin-layer chromatography carried out on 0.25 mm E. Merck silica gel plates (60F-254) with UV light, visualized by ceric sulfate solution. Flash column chromatography was performed on Silica Gel 60 N, purchased from Kanto Chemical Co. The T-shape mixer (Flom Co. Ltd., #9513, 19 mm × 28 mm × 8 mm, diameter 0.6 mm) was made of stainless steel and had a T-shape channel. The reaction tube (diameter 0.5 mm) was made of Teflon^®^. A Harvard Pump 11 Plus Single Syringe (HARVARD apparatus), a KDS 100 syringe pump, and a KDS 200 syringe pump (KD Scientific) were used to inject compounds into the T-shape mixers. The workup process included quenching of the reactions, liquid–liquid extraction, washing and drying, and was performed using a Zodiac CCX-1200 (Tokyo Rikakikai Co., Ltd.). Chromatographic separation was performed using a Purif^®^-α2 (Shoko Scientific Co., Ltd.).

#### Experimental details

**General procedure for the preparation of imidochlorides:** The mixture of *N*-(1-naphthyl)benzamide and SOCl_2_ (2 mL/mmol amide) was stirred at 65 °C for 4 h. The reaction mixture was concentrated in vacuo. The residue was used for the next reaction without further purification.

**General procedure for micro-flow synthesis of 1,2,3,4,5-pentaaryl-1,3,5-triazapenta-1,4-dienes 3b and 3c:** A T-shape mixer and reaction tube were immersed in a water bath (20 °C). Syringe pumps and a mixer were connected using a Teflon^®^ tube (diameter 0.25 mm). Imidochloride **4** was dried azeotropically with toluene. Aryl amine **5** or **9** (0.1 M), DIEA (0.7 M) and imidochloride **4** (0.1 M) were dissolved in CH_2_Cl_2_ under an argon atmosphere and were stored in syringes. Each solution was introduced to a T-shape mixer using the syringe pump. The mixed solution went through the reaction tube, and the resultant solution was poured into vigorously stirred saturated aqueous NH_4_Cl (1.5 mL) in CH_2_Cl_2_ (2 mL). After being stirred for several minutes, Et_2_O (30 mL) was added to the reaction mixture under vigorous stirring for 30 s. The aqueous layer was separated and added to saturated aqueous NaHCO_3_ (2 mL) after being vigorously stirred for 30 s. The aqueous layer was separated and brine (2 mL) was added followed by vigorous stirring for 30 s. After removing the aqueous layer, the organic layers were dried over Na_2_SO_4_ (10 g) and concentrated in vacuo. The residue was purified by column chromatography on silica gel (0% to 10% Et_2_O in hexane with 1% Et_3_N) to give 1,2,3,4,5-pentaaryl-1,3,5-triazapenta-1,4-diene **3b** or **3c**.

**1,3,5-Trinaphthyl-2,4-diphenyl-triazapenta-1,4-diene (3b):** ^1^H NMR (400 MHz, CDCl_3_) δ 8.22 (brd, *J* = 5.8 Hz, 1H), 8.15 (brs, 1H), 7.89 (brd, *J* = 5.4 Hz, 1H), 7.74–7.90 (m, 2H), 7.73 (brd, *J* = 6.8 Hz, 2H), 7.58 (brd, *J* = 7.8 Hz, 1H), 7.10–7.58 (m, 20H), 7.07 (t, *J* = 7.8 Hz, 1H), 7.39 (brd, *J* = 7.8, 1H), 6.64 (brs, 1H); ^13^C NMR (67.8 MHz, CDCl_3_) δ 156.2, 145.6, 136.1, 135.2, 134.7, 134.0, 130.1, 129.5, 128.8, 128.3, 128.2, 127.2, 126.3, 125.9, 125.8, 125.6, 125.2, 125.0, 123.6, 123.5, 123.0, 122.5, 121.6, 118.0, 115.7; FTIR (neat) 3055, 1624, 1572, 1529, 1495, 1393, 791, 771 cm^−1^; HRMS (ESI–TOF, *m*/*z*): [M + H]^+^ calcd. for C_44_H_32_N_3_, 602.2596; found, 602.2613.

**1,3-Dinaphthyl-2,4,5-triphenyl-triazapenta-1,4-diene (3c)**: ^1^H NMR (400 MHz, CDCl_3_) δ 8.29 (d, *J* = 6.4 Hz, 2H), 8.25 (d, *J* = 8.3 Hz, 2H), 7.78 (brd, *J* = 7.8 Hz, 1H), 7.73 (d, *J* = 7.8 Hz, 2H), 7.61 (d, *J* = 7.8 Hz, 1H), 7.36–7.60 (m, 9H), 7.40–7.20 (m, 2H), 7.16 (t, *J* = 7.8 Hz, 1H), 7.05–6.60 (m, 7H), 6.54 (brs, 1H), 5.71 (brd, *J* = 6.8, 1H); FTIR (neat) 3056, 1631, 1595, 1524, 1497, 1438, 1323, 775, 698 cm^−1^; HRMS (ESI–TOF, *m*/*z*): [M + H]^+^ calcd. for C_40_H_30_N_3_, 552.2440; found, 552.2435.

#### General procedure for the preparation of nickel complexes **12** and **13**

The following manipulations were performed under an inert atmosphere using standard glove box techniques. A solution of the prepared ligand **3b** or **3c** (1 equiv) and NiBr_2_(dme) (1 equiv) in dry CH_2_Cl_2_ (50 mL/mmol) was stirred for 4 h at room temperature. The obtained crude mixture was used for the polymerization without purification.

#### Procedure for the preparation of nickel complex **12** crystals

The following manipulations were performed under an inert atmosphere using standard glove box techniques. A solution of the prepared ligand **3b** (66 mg, 0.11 mmol) in 40 mL of dry CH_2_Cl_2_ was slowly added to NiBr_2_(dme) (34 mg, 0.11 mmol). The resultant mixture was stirred for 1 h at room temperature. Then, the stirring was stopped and the mixture was allowed to stand overnight at room temperature. Green colored needle-like crystals of **12**, suitable for X-ray analysis were obtained.

#### Procedure for the homopolymerization of ethylene

To a 2 L autoclave reactor, 1,000 mL of dry toluene and MMAO (500 equiv, 6.5 wt % in toluene) were added. The resultant mixture was heated to 60 °C, then the crude nickel complex (1 equiv) was injected under an ethylene pressure of 400 psig, which was fed continuously at that pressure over the course of the reaction. After being stirred for 0.5 h, ethanol was added to quench the polymerization, and ethylene was vented. The resultant mixture was collected, and concentrated in vacuo to afford a crude polymer. Polymerization activities were calculated from the mass of the crude polymer that was obtained.

#### Procedure for the copolymerization of ethylene with polar monomers

To a 2 L autoclave reactor, 1,000 mL of dry toluene and comonomers (500 equiv) along with MMAO (500 equiv, 6.5 wt % in toluene) were added. The resultant mixture was heated to 60 °C, then the crude nickel complex (1 equiv) was injected under an ethylene pressure of 400 psig, which was fed continuously at that pressure over the course of the reaction. After being stirred for 1 h, ethanol was added to quench the polymerization, and ethylene was vented. The resultant mixture was collected, and concentrated in vacuo to afford the crude polymer. Polymerization activities were calculated from the mass of the crude polymer that was obtained.

#### Procedure for the homopolymerization of propene

To a 2 L autoclave reactor, MMAO (500 equiv, 6.5 wt % in toluene) and 750 mL of propene were added. Then the crude nickel complex (1 equiv) was injected with nitrogen. The resultant mixture was heated to 60 °C. After being stirred for 1 h, ethanol was added to quench the polymerization, and propene was vented. The resultant mixture was collected, and concentrated in vacuo to afford a crude polymer***.*** Polymerization activities were calculated from the mass of the crude polymer that was obtained.

## Supporting Information

File 1 ^1^H and ^13^C NMR spectra.
