# Supplementary material for: Micro-flow synthesis and structural analysis of sterically crowded diimine ligands with five aryl rings
Source: Beilstein J Org Chem. 2013 Nov 1;9:2336–43. doi: 10.3762/bjoc.9.268 (PMC3869212; doi:10.3762/bjoc.9.268)
Supplement: File 1 — 1H and 13C NMR spectra. [file Beilstein_J_Org_Chem-09-2336-s001.pdf]

Supporting Information

for

Micro-flow synthesis and structural analysis of sterically crowded diimine ligands with five aryl rings

Shinichiro Fuse*[1], Nobutake Tanabe[1], Akio Tannna[2], Yohei Konishi[2] and Takashi Takahashi[1]

Address: [1]Department of Applied Chemistry, Tokyo Institute of Technology, 2-12-1, Ookayama, Meguro-ku, Tokyo, 152-8552, Japan and [2]Mitsubishi Chemical Croup, Science and Technology Research Center, Inc., 1000 Kamoshida-cho, Aoba-ku, Yokohama 227-8502, Japan

E-mail: Shinichiro Fuse* - sfuse@apc.titech.ac.jp

* Corresponding author

^{1}H and ^{13}C NMR spectra.

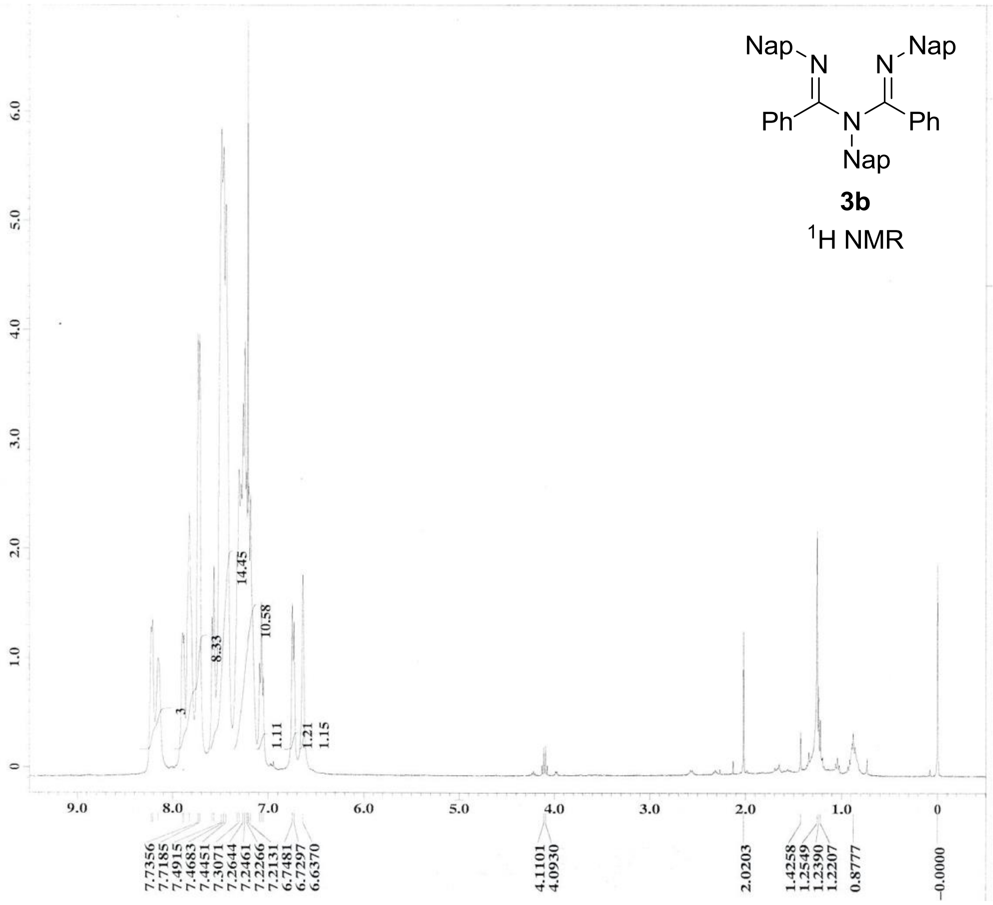

Nap
N
N
Nap
Ph
N
Ph
Nap
3b
1H NMR

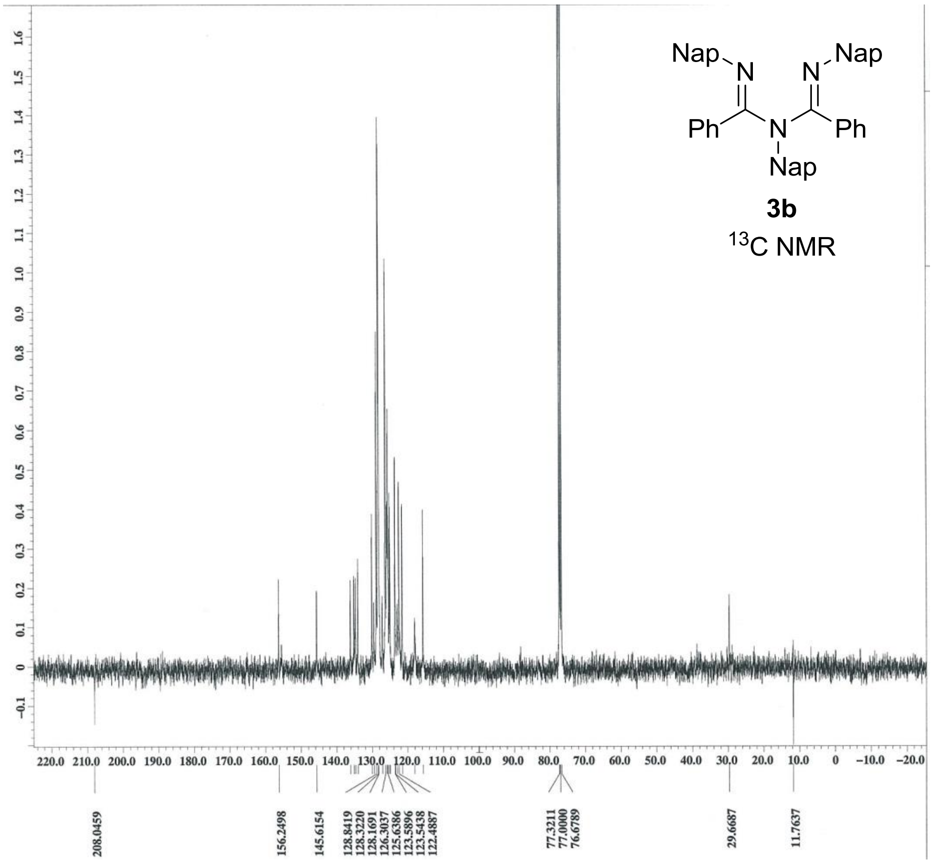
3b
13C NMR

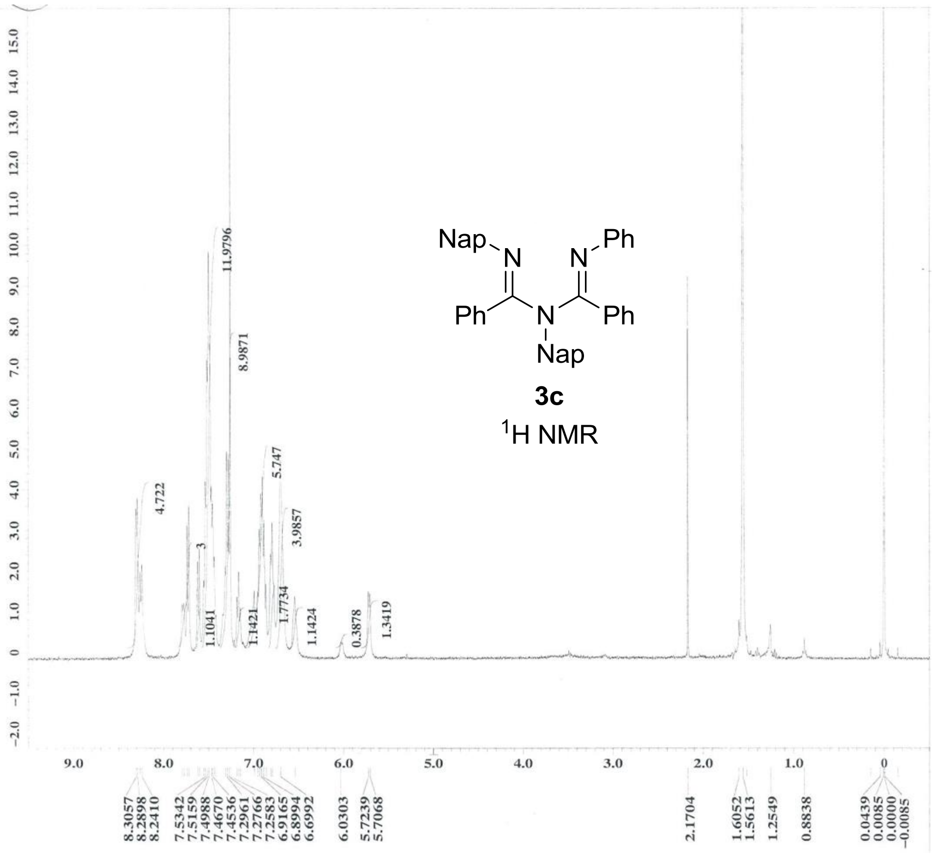
3c
1H NMR
